# The impact of Merowe Dam on *Simulium hamedense* vector of onchocerciasis in Abu Hamed focus - Northern Sudan

**DOI:** 10.1186/1756-3305-7-168

**Published:** 2014-04-04

**Authors:** Isam MA Zarroug, Arwa H Elaagip, Sara A Abuelmaali, Hanan A Mohamed, Wigdan A ElMubarak, Kamal Hashim, Tong Chor M Deran, Nabil Aziz, Tarig B Higazi

**Affiliations:** 1 Onchocerciasis Control/Elimination Programme, National Programme for Prevention of Blindness (NPPB), Federal Ministry of Health, Khartoum, Sudan; 2 Department of Parasitology and Medical Entomology, Faculty of Medical Laboratory Sciences, University of Khartoum, Khartoum, Sudan; 3 Department of Medical Entomology, National Public Health Laboratory, Federal Ministry of Health, Khartoum, Sudan; 4 National Programme for Prevention of Blindness (NPPB), Federal Ministry of Health, Khartoum, Sudan; 5 The Carter Center, Khartoum, Sudan; 6 Ohio University, Zanesville, Ohio, USA

**Keywords:** Onchocerciasis, Elimination, Merowe Dam, Abu Hamed focus, *Simulium hamedense*, Sudan

## Abstract

**Background:**

Abu Hamed, the northernmost onchocerciasis focus in the world, is located along the River Nile banks in the Nubian Desert. Hydroelectric dams can alter activity of black flies and may provide breeding sites for black fly. Merowe Dam, the largest hydropower project in Africa, was built west of Abu Hamed focus in 2009. The impact of the Dam on onchocerciasis and its black fly vectors in Abu Hamed focus was measured in this study.

**Findings:**

Entomological surveys for aquatic stages and adult *Simulium hamedense* were conducted before and after the inception of Merowe Dam in 2007/2008 and 2010/2011. There was no black fly breeding or adult activity in the previously known breeding sites upstream of the Merowe Dam with the western most breeding site found in AlSarsaf village near the center of the focus. No adult or aquatic stages of black flies were found downstream of the Dam.

**Conclusions:**

The artificial lake of the Dam flooded all the breeding sites in the western region of the focus and no aquatic stages and/or adult black fly activity were established in the study area upstream of the Dam. The Dam seems to have positive impact on onchocerciasis and its black fly vectors in Abu Hamed focus. These outcomes of the Merowe Dam might have contributed to the recently declared interruption of onchocerciasis transmission in Abu Hamed focus. Continuous entomological surveys are needed to monitor presence of black fly vectors and its impact on the disease.

## Findings

### Background

Onchocerciasis is endemic in most parts of tropical Africa, including most of East Africa between altitudes 15 N and 13S [1-3]. In Sudan, human onchocerciasis currently known in four main foci; Abu Hamed in Northern Sudan, Galabat in Eastern Sudan, Radom in Southern Darfur in the Southwest and Khor Yabus in Blue Nile region of the Southeast.

The Abu Hamed focus has been described along the River Nile in the Nubian Desert [4]. The focus is unique because it is isolated from other onchocerciasis foci in Sudan, for the arid nature of the surrounding Nubian Desert and its location as the northernmost onchocerciasis focus in the world [5,6]. The nearest focus is more than 600 km in the east of the country. Therefore, the focus is isolated by Nubian Desert, and if the disease is eliminated, there is little or no chance of being re-infected from other onchocerciasis foci. The control of onchocerciasis started with an annual community-based treatment with Ivermectin (CDTI) in 1998. In 2006, the Government of Sudan launched an onchocerciasis elimination policy and switched from annual to semi-annual (six months) CDTI. Comprehensive surveys showed a low level of transmission in 2007 [7] and complete interruption of the disease transmission in 2011 that was officially declared by the Sudan government in 2012 [8].

Black flies constitute serious public health and socio-economic problems as vectors of human onchocerciasis and biting nuisance in many rural parts of the world [9]. They breed in rivers in constant flow of fast-moving water where they attach to rocks and plants, and filter out suspended particles [3]. Human activities can lead to an increase of black fly numbers in an area. Structures such as concrete dams and concrete-lined stream channels provide excellent developmental sites for larvae and pupae of black fly species [10]. The black fly present at Abu Hamed focus, designated *S. hamedense*, represent a single monomorphoic population that is isolated geographically from all other *S. damnosum s.l.* populations in Africa. It is closely related, but separate, cyto-species of the complex [7]. In addition, *Onchocerca volvulus* parasites in Abu Hamed have been shown to carry a unique molecular pattern [11].

Building of a new Dam in Merowe town west of Abu Hamed focus was inaugurated in 2003. By its inception in 2009, the Merowe Dam has created an artificial reservoir that extends to Abu Hamed focus. Few studies have addressed impact of dams on transmission dynamics of onchocerciasis in African countries. New *Simulium* vector breeding sites were found near hydroelectric dams [12]; including infected black flies [13] providing links between construction of dams and the spread of black flies [14,15]. It has been suggested that onchocerciasis is the major environmental health problem around other dams in Africa [16,17]. In Sudan, few studies have highlighted the impact of dams on transmission dynamics of onchocerciasis. Increase of black fly bites with or without the disease were reported in the vicinity of Meridi and Roseires Dams indicating that dam construction may affect the dynamics and extent of onchocerciasis transmission [18,19].

This study was carried out to assess the impact of Merowe Dam on breeding and biting activities of onchocerciasis vectors in the context of the Onchocerciasis Control/Elimination Programme in the Abu Hamed focus in Northern Sudan.

## Methods

### Study area

**Figure 1:**
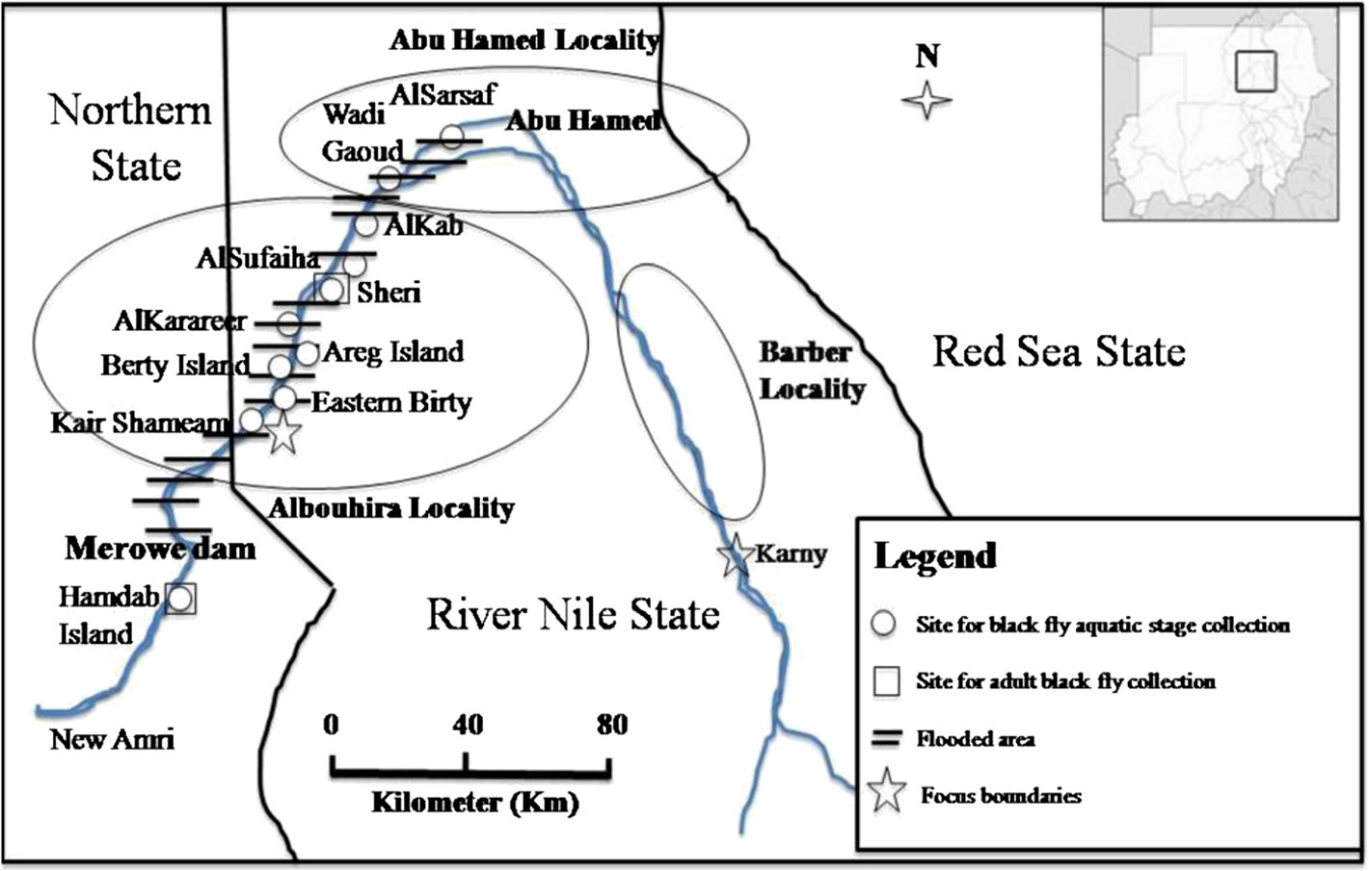
Map of the Abu Hamed focus showing Merowe Dam and the study sites.

The Abu Hamed focus extends for 300 km along the banks of River Nile in Barber, Abu Hamed and Albouhira localities of River Nile State and part of the Northern State adjacent to western boundary of Albouhira locality. The study area covered Abu Hamed locality (AlSarsaf and Wadi Gaoud) in the center of the focus, Albouhira locality (AlKab, AlSufaiha, Sheri, AlKarareer, Areg Island, Berty Island, Eastern Birty, Kair Shameam) west of Abu Hamed and Hamdab Island downstream the Dam outside the western end of the focus (Figure 1). With the exception of the Hamdab Island, these sites were previously known as breeding places of onchocerciasis vectors and disease infections have been reported from these sites including AlKab and Sheri areas.

The Merowe Dam is the largest contemporary hydropower project in Africa. It lies near Merowe town in northern Sudan at 18°40ʹ08ʺ N and 32°03ʹ01ʺ E, about 800 km downstream of Khartoum and 30 km downstream of the western end of Abu Hamed focus on River Nile in Sudan [8,20]. It has created a 200 km long artificial reservoir since its inception in 2009 (Figure 1). The formation of an artificial reservoir of Merowe Dam started at the peak of the flooding season of the River Nile in August 2008.

### Black fly collection

The aquatic stages of black flies were collected from vegetation in breeding sites of Sheri and AlSarsaf areas upstream of the Dam site and from Hamdab Island (outside the focus) during the 2007/2008 breeding season before the inception of the Dam and again during the 2010/2011 breeding season after the formation of an artificial reservoir (Table 1). Monthly biting rate surveys were carried out in Sheri area during 2007/2008 and 2010/2011 (Table 2) and in Hamdab Island during 2010/2011 (Table 2) to detect adult black fly activity. In each site, adult black flies were collected five days a month by four trained local collectors using the standard human landing capture method and rotating every three hours from 6:00 am to 6:00 pm. Hourly black fly collections were recorded and added to calculated flies per man-day [21]. Monthly biting rate was calculated as follows [21,22]:

**Table 1:** **The collection of aquatic stages of *Simulium hamedense *** **from study sites in Abu Hamed focus**

**Study site**	**Aquatic stages ( *Simulium hamedense *)**	
	**Before flooding of Merowe Dam (2007/2008)**	**After flooding of Merowe Dam (2010/2011)**
AlSarsaf	+ve	+ve
Wadi Gaoud*	+ve	-ve
AlKab	+ve	-ve
AlSufaiha	+ve	-ve
Sheri	+ve	-ve
AlKarareer	+ve	-ve
Areg island	+ve	-ve
Berty island	+ve	-ve
Eastern Birty	+ve	-ve
Kair Shameam**	+ve	-ve
Hamdab Island	-ve	-ve

^(*)^Last area affected by flooding of Merowe Dam.

^(**)^End of the Abu Hamed focus.

**Table 2:** Numbers and MBR of adult black flies in Sheri and Hamdab Island during 2007/2008 and 2010/2011

**2007/2008**	**Apr-07**	**May-07**	**Jun-07**	**Jul-07**	**Aug-07**	**Sep-07**	**Oct-07**	**Nov-07**	**Dec-07**	**Jan-08**	**Feb-08**	**Mar-08**
**Sheri - No. of flies**	4323	1578	797	105	0	0	0	354	1214	1891	2029	9599
**Sheri - MBR**	25938	9783.6	4782	651	0	0	0	2124	7526.8	11724.2	11768.2	59513.8
**2010/2011**	**Dec-10**	**Jan-11**	**Feb-11**	**Mar-11**	**Apr-11**	**May-11**	**Jun-11**	**Jul-11**	**Aug-11**	**Sep-11**	**Oct-11**	**Nov-11**
**Sheri - No. of flies**	0	0	0	0	0	0	0	0	0	0	0	0
**Hamdab Island – No. of flies**	0	0	0	0	0	0	0	0	0	0	0	0

$$\text{MBR}=\frac{\text{Number}\;\text{of}\;\text{flies}\;\text{caught}\times \text{Number}\;\text{of}\;\text{days}\;\text{in}\;\text{month}}{\text{Number}\;\text{of}\;\text{catching}\;\text{days}}$$

Ethical approval for this study was taken from Federal Ministry of Health, Sudan and informed consent was obtained from the collectors, and they were treated with Ivermectin semi-annually as part of the local population.

## Results

Black fly larvae were found in all studied breeding sites west of Abu Hamed town in 2007/2008. In contrast, no aquatic stages of black fly were found in the same sites in Albouhira locality and the western parts of Abu Hamed locality in 2010/2011 (Table 1) due to the flooding of breeding sites by Merowe Dam artificial reservoir. The last breeding site upstream of the Dam was found at AlSarsaf about 15 km west of Abu Hamed town (Figure 1, Table 1).

Adult black fly activity corresponding to previously described breeding season activity (between November and June) [7] was recorded monthly in Sheri area ranging from 105 to 9,599 collected flies and monthly biting rates of 651 to 59513.8 bites/person/month in July 2007 and March 2008 respectively. However, no black fly activity was detected in Sheri area after the flooding of the breeding sites by Merowe Dam artificial reservoir in 2010/2011 (Table 2). Inception of Merowe Dam has created new potential breeding places in Hamdab Island downstream of its site (Figure 1). However, no adults of black flies were detected in 2010/2011 (Table 2).

## Conclusions

Construction and operation of hydroelectric dams usually leads to changes in the physical and biological environment [15,16]. Adverse effects have more often than not, out-numbered the positive effect. Some of the negative impacts of hydropower include loss of vegetation, changes in riverine flow patterns and regimes, involuntary resettlement, health problems, loss of cultural values, marginalization of local people, inundating of valuable agricultural land, drought and severe reduction of flow downstream [16] in addition to creation of ideal and continuous breeding conditions for simulids [15].

Few studies have reported the impact of dams on onchocerciasis transmission in Africa and Sudan [12-19]. In this study, black fly breeding has not been established downstream of Merowe Dam. This finding is supported by study in Kpong dam in Ghana where the disease has been eliminated due to the reduction in the breeding conditions [16] and contradicted Jobin prediction that black flies will invade the spillways of the Merowe Dam [23]. Absence of black flies downstream of the Dam may be attributed to the distance between westernmost black fly breeding sites within Abu Hamed focus and the site of the Dam (>30 km) [8] and whether the local black flies are capable of crossing such a distance. It is also essential to note that plants and vegetation associated with black fly breeding in Abu Hamed e.g. *Digitaria ciliaris*[24] have not been found in the spillways of Merowe Dam. In addition, dams and their lakes could have unfavorable influence on the downstream river-bed fauna [25]. Although Hamdab Island is currently free from black fly breeding, continuation of entomological survey is highly recommended especially during months of high density of *Simulium* flies (January–April) downstream Merowe Dam.

The overall impact of Merowe Dam on Abu Hamed focus of onchocerciasis has been positive. The artificial reservoir of the Dam has submerged and eliminated black fly breeding sites west to Abu Hamed town, effectively shrinking the focus to a little over half of its original size. This finding supports the prediction of an overall decrease in river blindness in the area due to Merowe Dam [23]. However, the impact of the Dam on other vector-borne diseases like schistosomiasis and malaria in the area should be closely monitored.

Comprehensive entomological, parasitological and serological surveys of Abu Hamed focus after 13 years of annual and semi-annual CDTI provided proof for interruption of onchocerciasis transmission in the area in 2011 [8]. We believe that part of this success might be attributed to the positive effects of Merowe Dam on reduction of black fly breeding and activity described here. Extended monitoring of Merowe Dam spillways for black fly activity will ensure the integrity of the surveillance component of onchocerciasis elimination programmes in Abu Hamed.

## Competing interests

The authors declare that they have no competing interests.

## Authors’ contribution

IMAZ design the study, field work, collection of data, writing the manuscript; AHE data collection, data analysis, writing and revising the manuscript; SAA writing the manuscript; HAM and WAE field work, data collection; KH concept and idea of the work, TCMD and NA logistic and financial support; TBH writing and revising the manuscript. All authors read and approved the final version of the manuscript.
